# C1 hypertrophic lateral mass resection and occiput–C2 fusion

**DOI:** 10.3171/2020.4.FocusVid.20193

**Published:** 2020-07-01

**Authors:** Rajiv R. Iyer, Douglas L. Brockmeyer

**Affiliations:** Department of Neurosurgery, Division of Pediatric Neurosurgery, Primary Children’s Hospital, University of Utah, Salt Lake City, Utah

**Keywords:** Down syndrome, spinal cord compression, hypertrophy, fusion, pediatric

## Abstract

This case involved a 6-year-old boy with Down syndrome, left C1 lateral mass hypertrophy, C1–2 rotatory subluxation, and spinal cord compression. He presented after falling down some stairs at his home. Torticollis, dysphagia, and speech delay were noted on examination. Vascular imaging showed impingement on the left vertebral artery by the anomalous C1 lateral mass. Through a posterior approach, the hypertrophic C1 lateral mass was resected, and an occiput–C2 fusion was performed. Postoperatively, his torticollis and brainstem symptoms were resolved.

The video can be found here: https://youtu.be/1U0GLdw6c70

## Transcript

**0:28** Patient history. The case to present is of a 6-year-old boy with Down syndrome who presented after falling down the stairs. He was brought to an outside emergency room, where a CT and MRI was obtained. Further questioning from the parents found evidence of significant speech delay and chronic dysphagia, which had been noticed over many years.

His neurological examination was notable for torticollis and some neck discomfort, with a head tilt to the left. His cranial nerves were intact. He had full muscle power, normal ambulation, and full coordination.

**0:58** Preoperative imaging. Preoperative imaging showed significant anomalous hypertrophy of the C1 lateral mass, with a very abnormal relationship between the craniocervical joints. On the right-hand image, two lines are drawn to show the rotatory subluxation between the C1 and C2 bodies.

MRI scanning again showed significant C1 lateral mass hypertrophy with cord compression. The coronal images showed significant distortion of the cervicomedullary region from the C1 lateral mass. A CT angiogram was obtained. The right-sided image shows significant stenosis of the left-sided vertebral artery with an anomalous course through the hypertrophic C1 lateral mass. The right vertebral artery was diminutive, which is seen on the left-hand image.

**1:45** Surgical procedure. The surgical procedure was through a standard midline suboccipital incision. On the right-hand side is the occiput; on the left-hand side is the subaxial region. We dissected down toward the C1–2 region, and we encountered significant scar tissue formation between the C1 and C2 joints. Here, we’re approaching the C2 nerve root on the left-hand side, and we are carefully dissecting it out under the microscope. Once we had identified it, we coagulated and divided the C2 nerve root without incident.

At this point, we freed up more soft tissue underneath the C2 nerve root and could identify the hypertrophic C1 lateral mass. We carefully dissected around the C1 lateral mass both inferior and superior in order to delineate the space between the dura and the bone.

**2:41** Drilling hypertrophic C1 lateral mass. Here the microscope is turned, with superior at the top, inferior at the bottom, and left and right. At this point, we began drilling out the hypertrophic C1 lateral mass in an inside-out approach. We drilled out the inside and then eggshelled the bone and very carefully retracted the dura using a small Penfield dissector shown here. Small curettes were used to develop the plane between the dura and the bone, and we slowly worked our way down toward the front of the spinal canal. Rongeurs and drills and Kerrisons were used to remove the bone without difficulty. You can see the C1 lateral mass rocking as we’re working, which is evidence of the ultimate instability of the craniocervical region and the necessity for fusion.

Here, we use a stereotactic navigation probe in order to identify the area in which we’re working. As you can see, we are past the area of compression of the dura, and here we’re retracting laterally on the spinal cord to ensure that full decompression of the spinal cord is achieved.

**3:51** Postoperative course. Postoperative imaging showed complete resection of the C1 lateral mass on both axial and coronal images. Here, a plain film shows the occiput–C2 fusion construct. The patient did well postoperatively and was discharged in good condition on postoperative day 2 in a cervical collar. At 2-month follow-up, his torticollis was completely resolved, and his neck mobility was normal. On closer questioning with the parents, they stated that he had progressed significantly in his eating, speech ability, and fluency, and had remained at full strength (Hankinson et al., 2010; Kennedy et al., 2016; Mazur et al., 2014).
